# Isoniazid causes heart looping disorder in zebrafish embryos by the induction of oxidative stress

**DOI:** 10.1186/s40360-020-0399-2

**Published:** 2020-03-12

**Authors:** Jie Ni, Hongye Wang, Xiyi Wei, Kangjie Shen, Yeqin Sha, Yuxiang Dong, Yimei Shu, Xiaojie Wan, Jingwen Cheng, Fang Wang, Yihai Liu

**Affiliations:** 1 Department of Emergency, Affiliated Nanjing Drum Tower Hospital of Nanjing University Medical School, 321 Zhongshan Road, Nanjing, 210008 Jiangsu Province China; 2 The First Clinical Medical School, Nanjing Medical University, Nanjing, China; 3 Department of Urology, The First Affiliated Hospital of Nanjing Medical University, Nanjing, China; 4 Clinical School of Imaging, Nanjing Medical University, Nanjing, China; 5 The Medical School of Pediatrics, Nanjing Medical University, Nanjing, China; 6 Department of Cardiology, Nanjing Drum Tower Hospital, Clinical College of Nanjing Medical University, Nanjing, China

**Keywords:** Isoniazid, Heart looping disorder, Oxidative stress

## Abstract

**Background:**

The cardiotoxicity of isoniazid on zebrafish embryos and its underlying mechanism is unclear.

**Methods:**

Here, we exposed zebrafish embryos at 4 h post-fertilization to different levels of isoniazid and recorded the morphology and number of malformed and dead embryos under the microscope.

**Results:**

The high concentration of isoniazid group showed more malformed and dead embryos than the low concentration of isoniazid group and control group. The morphology of the heart and its alteration were visualized using transgenic zebrafish (*cmlc2*: GFP) and confirmed by in situ hybridization. The negative effects of isoniazid on the developing heart were characterized by lower heart rate and more heart looping disorders. Mechanistically, PCR showed decreased expression of heart-specific transcription factors when exposed to isoniazid. Oxidative stress was induced by isoniazid in cardiomyocytes, mediated by decreased activities of catalase and superoxide dismutase, which were rescued by scavengers of reactive oxygen species.

**Conclusion:**

In conclusion, this study demonstrated that isoniazid led to heart looping disturbance by the downregulation of cardiac-specific transcription factors and induction of cardiomyocyte apoptosis.

## Background

Tuberculosis is a highly infectious disease that has a high prevalence in China [1]. The common anti-tuberculosis drugs include isoniazid, rifampicin, ethambutol, and pyrazinamide, of which isoniazid is regarded as an irreplaceable first-line anti-tuberculosis drug [2, 3]. Isoniazid, also known as isonicotinylhydrazide, was first introduced in 1995 and is still used today for its high efficacy against tubercle bacillus [4]. Isoniazid inhibits the growth and reproduction of tubercle bacillus by the production of reactive oxygen species (ROS) and reactive nitrogen species [5]. Furthermore, isoniazid destroys bacterial cell walls by the prevention of the synthesis of branching acids, mediated by the activation of the KatG enzyme on its entry into tubercle bacillus. The mutation of KatG is associated with isoniazid resistance in tubercle bacillus [5, 6].

In addition to the extremely strong anti-tuberculosis effect, side effects of isoniazid have been observed clinically [3, 7]. One common side effect is liver damage, as approximately 20% of patients have increased AST (aspartate aminotransferase) and ALT (alanine aminotransferase) levels in the blood after administration of isoniazid [8] and approximately 1% of patients have severe hepatocyte damage [9]. However, some studies discovered that isoniazid can lead to skin rash [10] and endocrine disturbance [11]. Although isoniazid is regarded as a Pregnancy Category C drug by the Food and Drug Administration [12], some studies have demonstrated potential toxic effects to a fetus. Zhang et al. discovered that isoniazid affects liver development in zebrafish embryos and causes liver injury by the construction of *fabp10a:mcherry* transgenic zebrafish [13]. Additionally, the abnormal development of the nervous system, skull bone, and cartilage in a zebrafish embryo due to isoniazid has been demonstrated in vitro and in vivo [14]. However, there is no definitive report in regard to whether isoniazid has toxic effects on the developing heart of a zebrafish embryo.

According to epidemiological statistics, the occurrence of congenital heart disease is approximately 3–4% in infants [15–17] and is now one of the most common causes of neonatal death [18]. The types of congenital heart disease include ventricular septal defect, atrioventricular valve defect, transposition of the aorta, and constriction of the aorta. The occurrence of congenital heart disease is attributed to many factors, such as genetic factors, environmental factors, pathogenic microorganism infections, and drug toxicity, among which drug toxicity plays an important role in cardiac development defects [19, 20]. For example, tilmicosin [21], doxorubicin [22], cisplatin [23], and diclofenac sodium [24] have been reported to induce cardiotoxicity by the dysregulation of oxidative stress. However, the teratogenicity and cardiotoxicity of isoniazid on fetal heart development are rarely reported. The zebrafish is a widely used animal model due to short spawning periods and transparent embryos. Zebrafish embryos are often used to investigate drug toxicity and the molecular mechanisms of development [25, 26]. In our study, we utilized *cmlc2*:GFP heart transgenic and wild type zebrafish embryos. We observed that isoniazid disturbed the cyclization process during heart development of zebrafish embryos and caused congenital cardiac malformation. Mechanically, it was mediated by inducing oxidative stress and cardiomyocytes apoptosis and alleviation of oxidative stress can protect cardiac development under the exposure to isoniazid.

## Materials and methods

### Zebrafish husbandry

The wide type zebrafish (AB strain) was purchased from the Institute of Hydrobiology of the Chinese Academy of Science (Wuhan, China) and transgenic zebrafish (AB *cmlc2*:GFP) were gifted by Dr. Lou in the Nanjing University Animal Model Institute. These were kept in the Animal Model Center in the Jiangsu Province Key Laboratory of Human Functional Genomics. Recycled water at 28 ± 0.5 °C and a 14 h light cycle per day were set to mimic the circadian rhythm. Zebrafish were fed with brine shrimp twice a day. At 17:00, we transferred the male and female adult zebrafish into the mating tank in a ratio of 2:1 and segregated them with a partition. The next morning, we moved the partition at 9:00 and the mating process lasted for 2 h. After spawning, the embryos were collected in clean fish water. The experiment protocols were approved by the Animal Ethics Committee of Nanjing Medical University.

### Chemical exposure experiment

We used 0 mM, 0.01 mM, 0.04 mM, 0.16 mM, 0.64 mM, 2.56 mM, and 10.24 mM doses of isoniazid (I3377-50G; Sigma) for the dose-effect assay according to the therapeutic range of 3–5 μg/mL (0.01–0.04 mM) of isoniazid [27]. No precipitation was observed at room temperature 25 °C. The embryos were cultured in sterile water composed of 137 mM/L NaCl, 0.54 mM/L KCl, 0.025 mM/L Na_2_HPO_4_, 0.044 mM/L KH_2_PO_4_, 1.3 mM/L CaCl_2_,1 mM/L MgSO_4_, and 0.42 mM/L NaHCO_3_. N-acetyl-L-cysteine (NAC; A7250-50G; Sigma), a ROS scavenger, was added (1 mM) in advance to clean out ROS in the embryos in the NAC group for the rescue experiments. After 4 h post-fertilization (hpf), 80 well-grown embryos in each group were selected under the microscope and kept in one 100 mm plate (CORNING; Corning; United States) for embryo culture. The culture water that contained isoniazid was changed every 12 h and these embryos were kept in the incubator at 28 °C and 5% CO_2_. We recorded the number and morphology of malformed and dead embryos and removed dead embryos every 12 h after exposure. Each chemical exposure treatment was performed in triplicate.

### Morphology observation

We observed the morphology of embryos in different stages of development (24 hpf, 48 hpf, and 72 hpf) under white light with a fluorescent microscope (Nikon, Tokyo, Japan). In each stage, the total number of dead, unhatched, and malformed embryos was recorded. The indication of embryo death was defined based on opacity, egg clotting and stopping of heartbeat. We randomly selected 20 larvae and recorded the heart rate in 1 min in each experimental group. A transgenic zebrafish strain (*cmlc2*:GFP), where a green fluorescent protein gene was inserted under the promoter of a cardiomyocyte-specific gene of cardiac myosin light chain 2 (*cmlc2)*, was applied to observe the change in morphology during heart development. All experiments were performed in triplicate.

### Real-time PCR

After 48 h of chemical exposure, the total RNA of 20 larvae were isolated using Trizol reagent (Thermofisher, Waltham, United State). The total RNA in each group were reverse transcribed to produce cDNA with a TaKaRa RT reagent kit (TaKaRa, Japan). The primers of cardiomyocyte-specific genes were synthesized by Invitrogen (Shanghai, China) and their sequences are listed in Table 1. Real-time PCR was performed in triplicate with a mixture of SYBR green, primers, cDNA, and RNase-free water in an appropriate ratio. A housekeeping gene, 18 s ribosomal RNA, was used for normalization.

**Table 1:** Sequences of primers in RT-PCR

Target Gene	Primer Sequences
18 s	Forward:5’TCGCTAGTTGGCATCGTTTATG 3′ Reverse:5′ CGGAGGTTCGAAGACGATCA 3’
Amhc	Forward:5’ AAGGTAAAATCCTACAAACGTTCGG 3′ Reverse:5′ CAAACAAATCAAAGTGCGATTGCAC 3’
cTNT	Forward:5’ GTCTGCACTTCGGCGGTTACA 3′ Reverse:5’AGGTAAAATCTATATTGTTCAGTGAAATCTAACCG3’
Vmhc	Forward:5′ ACATAGCCCGTCTTCAGGATTTGG 3′ Reverse:5′ GAGAGAAAGGCAAGCAAGTACTGG 3’
Cmlc2	Forward:5’ AGACCCAGAGGAAACCATCC 3′ Reverse:5′ TTGGGTCATTAGCAGCCTCT 3’
Tbx5	Forward:5’CGCTATAAATTCGCCGATAACAA 3′ Reverse:5′ AGACACCAGTTGCCTCATCCAG3’
Hand2	Forward:5’ GGACATTCTGGACAAAGATGAA 3′ Reverse:5′ GCCAACCAGTTCTCCCTTTA 3’
Gata4	Forward:5’ CCAGACACACACCAGCTCTACAC 3′ Reverse:5′ ATCAGGCTGTTCCACACTTCACT 3’

### Acridine orange (AO) staining

Acridine orange, a permeable nucleic acid-selective fluorescent dye (Thermo Fisher Scientific), was used to assess the apoptosis level of cardiomyocytes during the development of zebrafish that were exposed to different doses of isoniazid. Ten randomly selected embryos at 48 hpf were stained with AO for 30 min and washed with phosphate-buffered saline (PBS). A fluorescence microscope (Nikon, Tokyo, Japan) was used for photography.

### ROS staining

2′,7′-Dichlorodihydrofluorescein diacetate (DCFH-DA; Beyotime Biotechnology, China), a permeable and sensitive probe, was used for the detection of ROS in this study. We randomly selected 10 embryos at 48 hpf and stained them for 40 min with 100 μM of DCFH-DA diluted in PBS. After this was washed out with PBS for 10 min, all groups were photographed under a fluorescence microscope under a fluorescein isothiocyanate channel. All steps were finished in the absence of light.

### Antioxidant enzyme activity assay

In each group, 20 zebrafish embryos were lysed in radioimmunoprecipitation assay buffer and cocktails. Commercial kits (Beyotime Biotechnology, China) were used to detect the activity of two important antioxidant enzymes, superoxide dismutase (SOD) and catalase (CAT). We also evaluated the level of malondialdehyde (MDA), another marker for oxidative stress. Finally, the level of ROS was measured using a commercial kit (Beyotime Biotechnology, China). All experiments were performed in triplicate.

### In situ hybridization

Embryos for in situ hybridization were exposed to propylenethiourea, a chemical reagent that removes the melanin from larvae. After that, we fixed the embryos in 4% paraformaldehyde and preserved them at 4 °C. This experiment lasted for 3 days. On the first day, we used 25, 50, 75, and 100% ethanol for dehydration and a protease kinase to digest tissues for 10 min. Next, 0.1 mol/L triethanolamine with 25% acetic anhydride was added to acidized the embryos and a nuclear acid probe of *cmlc2* for hybridization was added at 60 °C. On the second day, we used 2 × saline sodium citrate (SSC) and 0.2 × SSC buffer to wash out the residual probes. Then, we added an antibody against digoxin overnight at 4 °C. On the last day, we washed out the antibody with 1% maleic acid buffer and dried the embryos with BM purple for 10 min. Finally, we observed the dried embryos on a plate with 5% agar powder with an upright microscope (Olympus, Japan) under white light.

### Pericardial area measurement

After we captured the images of embryos under the white light with a microscope (Nikon, Tokyo, Japan) using a 1.5 × lens, the pericardial area was measured using NIH Image J 1.44 software (http://rsb.info.nih.gov/nih-image/). Boundaries and area of pericardial area were traced and calculated by the computer. The experiment was conducted with three replicates (*n* = 3) and each group contained 50–80 embryos.

### Statistical analysis

Continuous variables are presented as mean and SD, whereas categorical variables were presented as number and percentage. For continuous variables, the difference between all groups was compared with a one-way ANOVA test. For categorical variables, a chi-square test was used. *P* < 0.05 was a significant statistical difference. An asterisk denotes a statistical significance between two groups (^*^P < 0.05, ^**^P < 0.1, ^***^P < 0.001). All statistical tests were completed in SPSS 22.0.

## Results

### The effect of isoniazid on mortality and hatchability of embryos

**Fig. 1:**
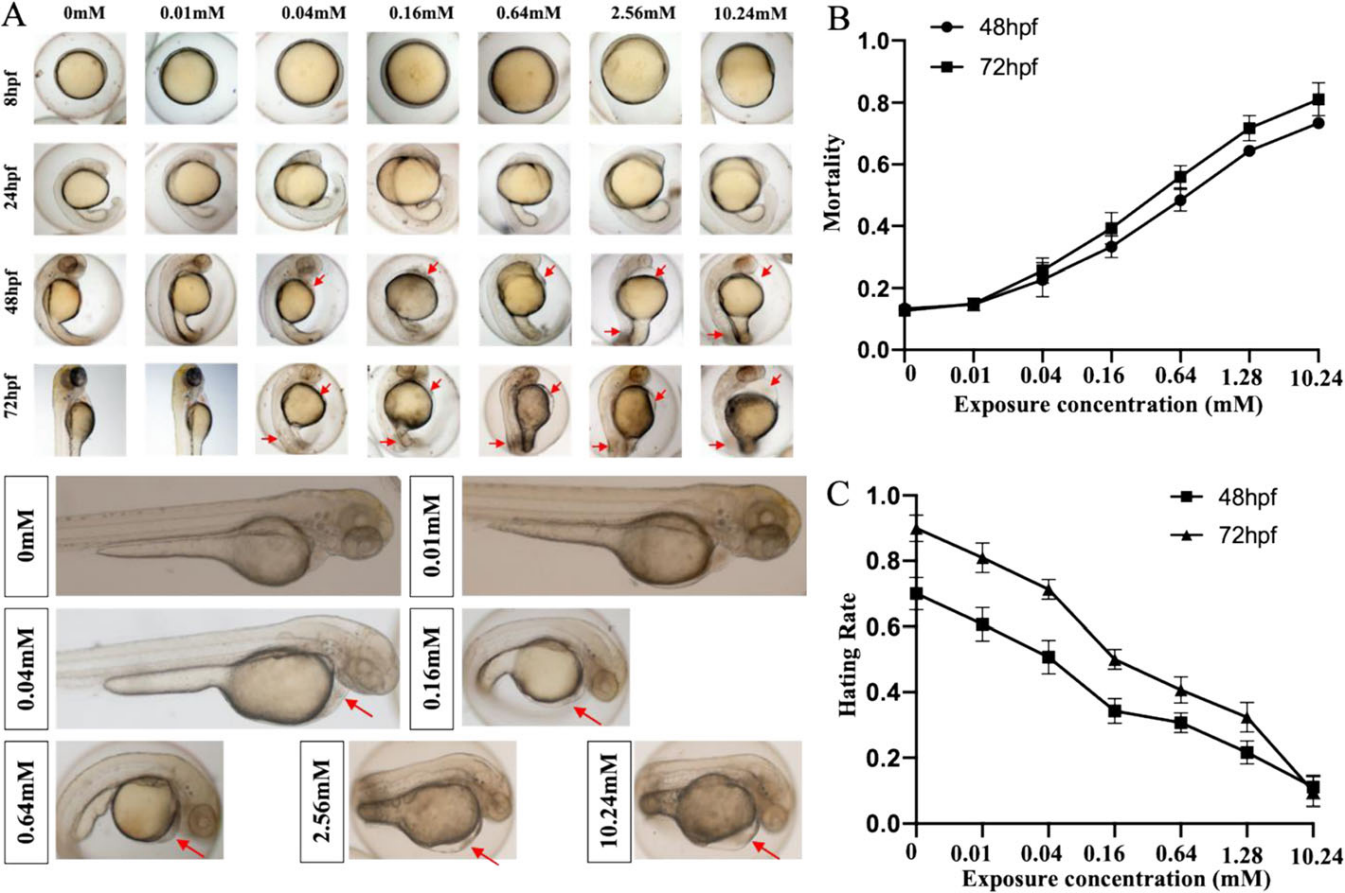
Morphology alteration (**a**), mortality (**b**) and Hatching rate (**c**) of zebrafish embryos exposed to different concentrations of isoniazid at 48 and 72hpf. The red arrows represented some typical deformities in the embryo development like yolk sac edema and spinal curvature due to the abnormal development of somatic ganglia.^*^P < 0.05, ^**^P < 0.1, ^***^P < 0.001

In our experiment, the mortality and hatching rate of zebrafish embryos exposed to different concentrations of isoniazid (0 mM, 0.01 mM, 0.04 mM, 0.16 mM, 0.64 mM, 2.56 mM, and 10.24 mM) at different time points were recorded (Fig. 1b, c). In the treated group, the mortality of embryos increased and hatchability decreased compared to the unexposed control group, and both were associated with dose. Additionally, although there was no difference in morphology at the early stage, we observed that zebrafish embryos had abnormal development of somatic ganglia and yolk sac edema at 48 and 72 hpf (Fig. 1a).

### The effect of isoniazid on heart development of embryos

**Fig. 2:**
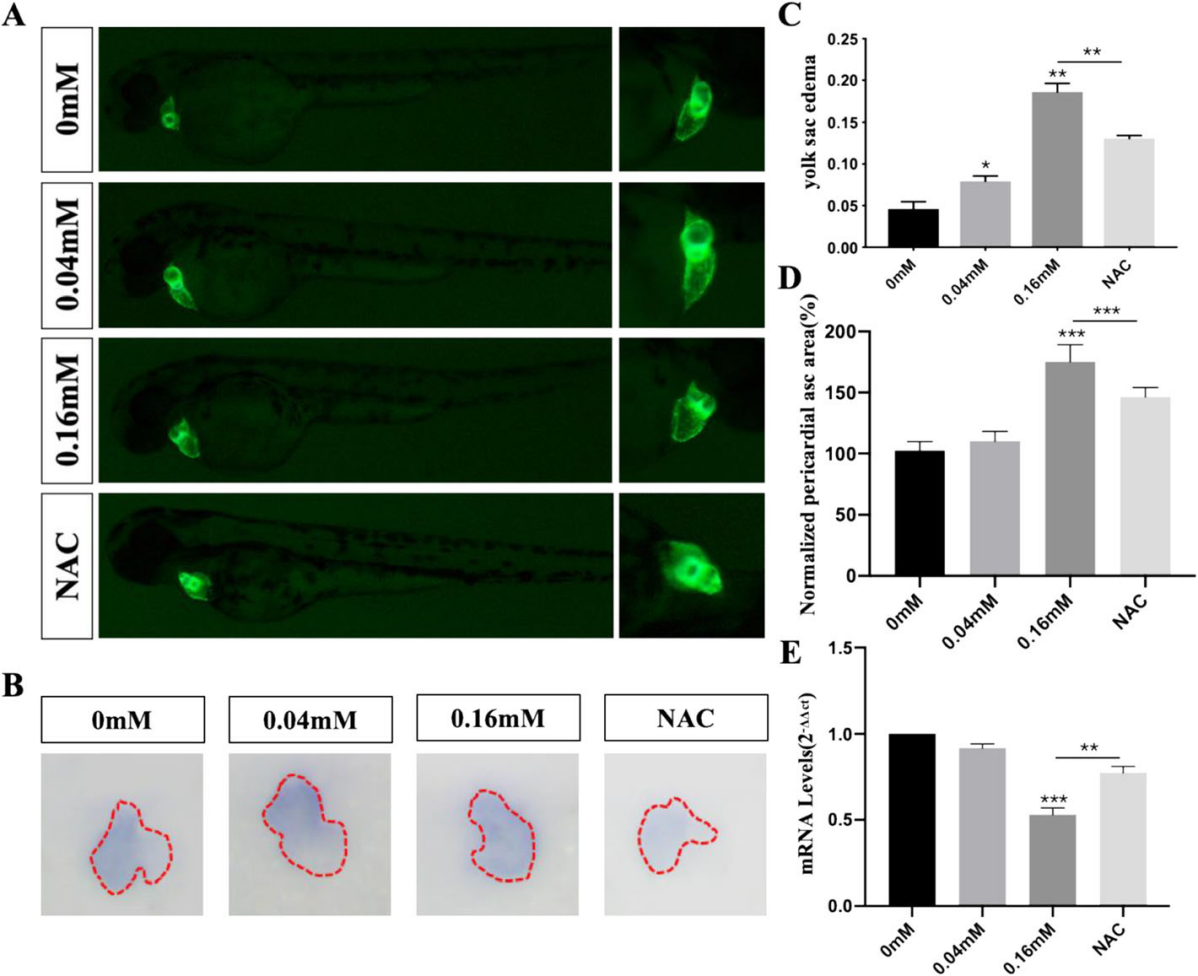
The alteration in the cardiac morphology at 48hpf by using transgenic strain zebrafish (Cmlc2-GFP) The red lines represented the outline of the heart (**a**). The results of in situ hybridization by using a cmlc2 probe (**b**). Isoniazid lead to yolk sac edema during the cardiac development (**c**). Measurement of Pericardial asc area and normalized to 0 mM groups (**d**). Expression level of cTNT (Cardiac Troponin T) at 48hpf in 0 mM, 0.04 mM, 0.16 mM and NAC groups (**e**). ^*^P < 0.05, ^**^P < 0.1, ^***^P < 0.001

**Fig. 3:**
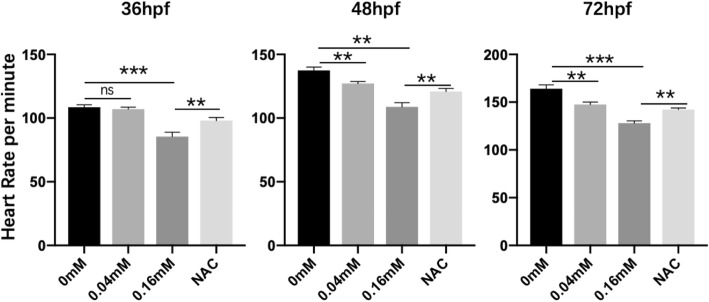
The heart rate of zebrafish embryos recorded at different times in control, 0.04 mM, 0.16 mM and NAC groups. ^*^P < 0.05, ^**^P < 0.1, ^***^P < 0.001, ns means no significantly different

**Fig. 4:**
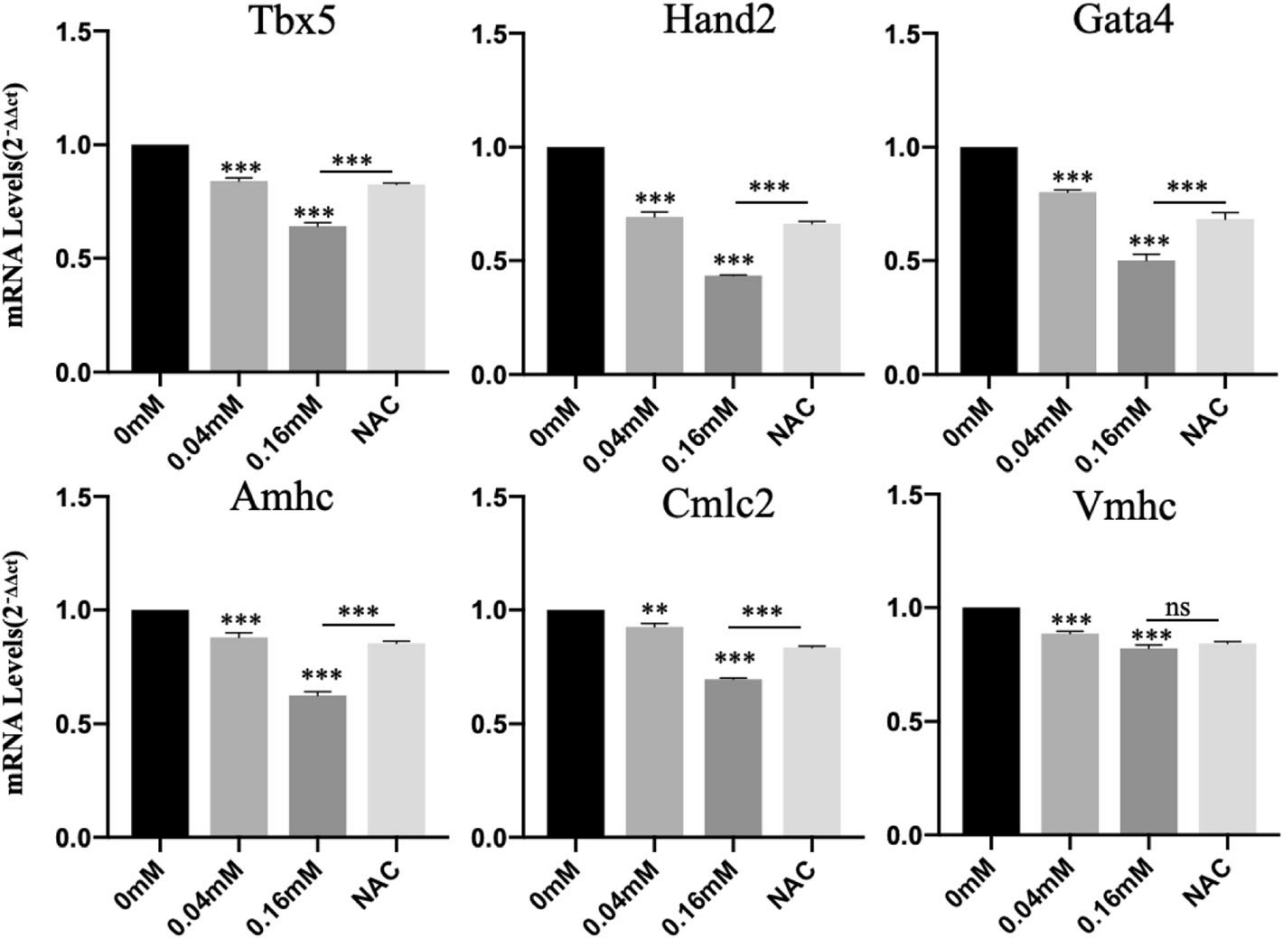
The expression of four cardiac-specific transcript factors in zebrafish embryos after 48 hpf of isoniazid exposure. ^*^P < 0.05, ^**^P < 0.1, ^***^P < 0.001, ns means no significantly different

The heart development of zebrafish embryos underwent a series process including cardiogenic specification and differentiation, formation of bilateral heart field, myocardia tube rotation, cardiac looping and chamber ballooning, and atrioventricular valve formation [28]. We observed cardiac morphological changes at 48 hpf using *cmlc2*:GFP transgenic zebrafish. The treatment groups developed cardiac cyclization disorders during 48 hpf, unlike the control group, which were alleviated by antioxidative NAC (Fig. 2a). The results of the in situ hybridization of *cmlc2* also indicated that isoniazid caused cardiac cyclization disorders (Fig. 2b). Some of these embryos had yolk sac edema, of which the proportion in treatment groups was higher than that in the control group (Fig. 2c) and the pericardial areas were decreased in the isoniazid treated groups compared to those in the control group (Fig. 2d). The downregulated expression level of cardiac troponin T (cTNT) indicated the cardiotoxicity of isoniazid (Fig. 2e). We discovered that isoniazid decreased the heart rate of zebrafish larvae and that this could be rescued by NAC treatment (Fig. 3). Furthermore, severe arrhythmia occurred in the 0.04 mM and 0.16 mM groups (Data not shown). Several key transcriptional factors including Tbx5, Gata4, and Hand2, which control cardiac cyclization during cardiac development, showed decreased expression in embryos treated with isoniazid (Fig. 4). We also investigated marker genes associated with ventricular and atrial development, such as *amhc* (atrial myosin heavy-chain), *vmhc* (ventricular myosin heavy-chain), and *cmlc2* (cardiac myosin light chain 2) (Fig. 4). These experimental results suggested that isoniazid caused embryo development deficiency, which may be mediated by the inhibition of heart looping.

### Oxidative stress in cardiomyocytes induced by isoniazid

**Fig. 5:**
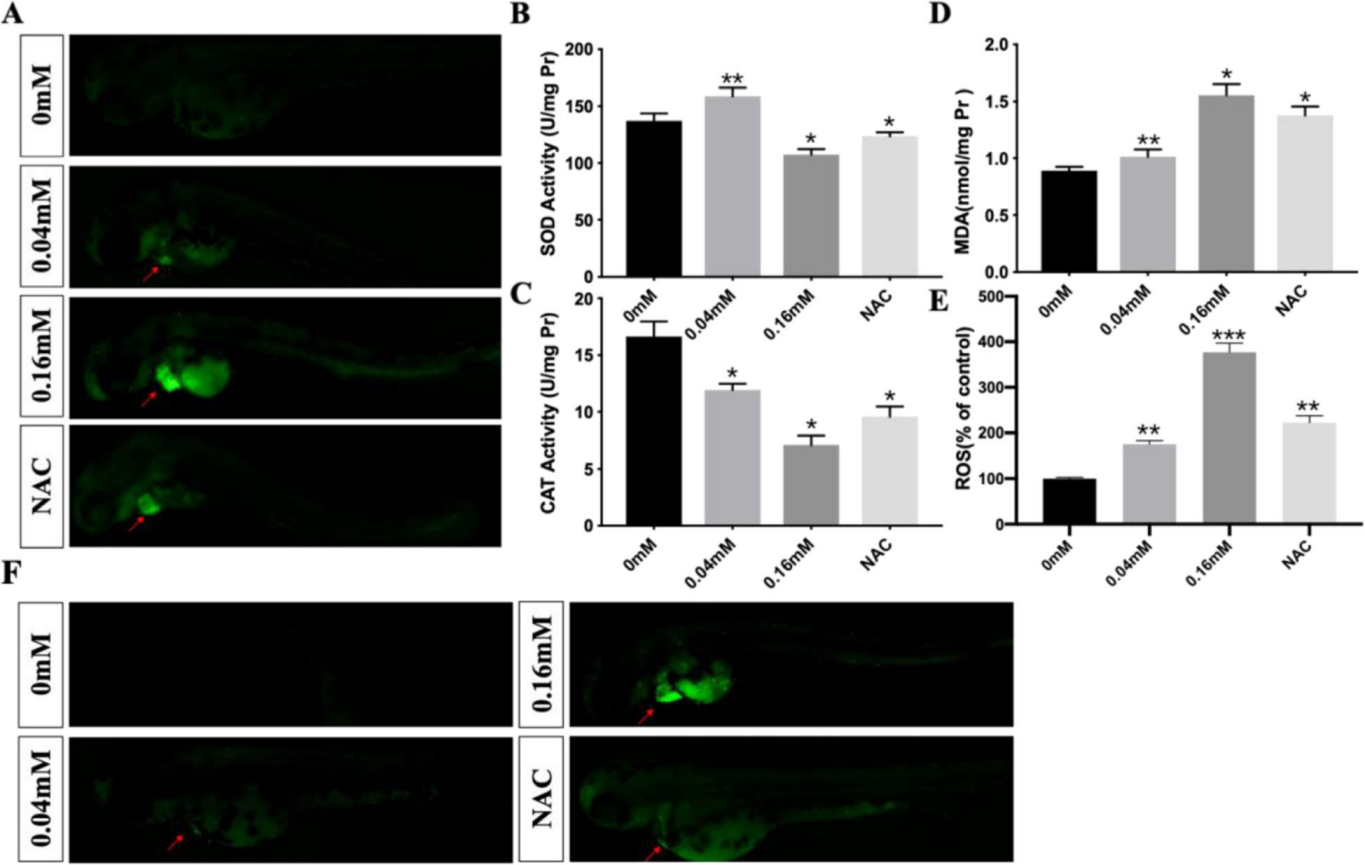
The ROS level in control, 0.04 mM, 0.16 mM and NAC groups (**a**). The activity of CAT (**b**) and SOD (**c**) induced by isoniazid. The concentration of MDA (**d**), ROS (**e**) and apoptosis level (**f**) in different treated groups. The unit of SOD and CAT were ul/mg protein while MDA nmol/mg protein. The concentration of ROS was normalized to 0 mM groups. The red arrows represented the pericardial areas. ^*^P < 0.05, ^**^P < 0.1, ^***^P < 0.001, ns means no significance

Previous studies have reported that isoniazid causes the accumulation of ROS in cells, and high ROS can cause heart looping deficiency during cardiac development. To explore the mechanism behind heart looping defects, we used an ROS fluorescence probe to label 48 hpf zebrafish embryos and performed an MDA assay. We demonstrated that there was serious oxidation stress in the hearts of zebrafish embryos in the treated groups and that the effect was linked to isoniazid concentration (Fig. 5a, d, e). We also detected the activity of two important antioxidant enzymes, SOD and CAT, and both enzymes had lower activity in the 0.16 mM treated group (Fig. 5b, c) than that in the other groups. However, a higher activity of SOD enzyme was observed in the 0.04 mM treated group, which indicated potential compensatory effects in the embryos for scavenging ROS (Fig. 5b). An antioxidant was used in the rescue experiment. We discovered that NAC effectively reversed the heart development deficiency caused by isoniazid (Figs. 2a, b, c, 3, 4). These results may explain how isoniazid causes heart looping deficiency and how this depends on oxidative stress. Finally, we observed that isoniazid induced cardiac cell apoptosis by AO staining and a ROS scavenger rescued cells from apoptosis (Fig. 5f).

## Discussion

As a first-line anti-tuberculosis drug, isoniazid plays an irreplaceable role in the treatment of tuberculosis [2]. Liver toxicity and gastrointestinal reaction were predominant side effects observed during clinical drug use and several cases indicate that isoniazid causes cardiotoxicity [29, 30]. In our study, we discovered that isoniazid caused a deficiency in cardiac development via a ROS dependent manner. High levels of isoniazid reduced the hatch and survival rates of zebrafish embryos, which was linked to heart development using heart-specific transgenic zebrafish. By combining ROS and AO staining, and oxidant enzyme and antioxidant enzyme assays, we demonstrated that isoniazid induced oxidative stress and cardiomyocyte apoptosis during heart development. It has been reported that isoniazid causes oxidative stress and apoptosis in tumor cell line Hep3B, which may be related to its high metabolic demand and strong proliferative ability [31]. Thus, the differential response to isoniazid between neonatal myocytes and adult cardiomyocytes is accounted for. Embryonic cells have a strong proliferative capacity, whereas mature cardiac cells have a more mature antioxidant system.

During embryonic development, immature cells are stem-like and have a strong ability for proliferation and differentiation. Therefore, they can be easily affected by the external environment and drugs via alterations to transcriptional regulation. The heart is the first organ to form during embryonic development. It forms in the following stages: the formation of cardiogenic zones from cardiac progenitor cells, with the first cardiogenic zone and the second cardiogenic zone [32, 33]; the appearance of a linear cardiac tube due to separation and migration of cardiogenic zones at the proto-intestinal stage, with the heart starting to beat; the looping of the linear cardiac tube; the arising of primitive atrium and ventricle structure from endocardial endothelial cells, with undergoing endothelial mesenchymal transformation into endocardial cushions and a heart comes into being after cardiac cell remolding [34–36]. Heart development is regulated by a combination of transcription factors, in which downregulation leads to defects in heart development, teratogenicity, and even death of embryos [37]. In our experiment, transcription factors related to cardiac development were downregulated in the high concentration group, as explained by the cardiotoxicity of isoniazid at the molecular level.

ROS are a series of chemicals with strong oxidative capacities that are produced during cell metabolism, including superoxide radicals, hydroxyl radicals, and hydrogen peroxides. A high concentration of ROS induces oxidative stress and mitochondrial dysregulation in cells, which causes the oxidation of lipids, proteins, and nucleic acids and destroys their biological functions. ROS are signal molecules and play important roles in the process of embryonic development. ROS participate in the development of the nervous system, the differentiation and formation of the nerve, and the development of lung and vascular tissue [38]. Recently, an article reported that high levels of ROS lead to heart looping disorder during heart development [39, 40]. There are also many reports that environmental poisons and drugs such as doxorubicin [41], chlorpyrifos [42], and dinitrobenzene cause abnormal development of various organs by the induction of ROS accumulation. In our study, we observed similar consequences, that ROS was highly concentrated in the heart and cardiac looping was impaired in the treatment group, which suggested that isoniazid caused cardiac dysplasia by the upregulation of ROS. In the high concentration group, the atrial and ventricle became smaller than those in the middle concentration group and the control group, which may have been due to apoptosis and cell cycle arrest of developing cardiomyocytes caused by ROS. However, our study have some limits. First, the molecular mechanism and signaling pathway that underlie the cardiotoxicity of isoniazid need to be further investigated. Second, the chorion effect may lead to the low concentration of drug exposure, which need a future experiment by utilizing dechorionated embryos [43].

## Conclusions

We observed that isoniazid disturbed the cyclization process during heart development of zebrafish embryos and caused congenital cardiac malformation. Mechanically, it was mediated by inducing oxidative stress and cardiomyocytes apoptosis and alleviation of oxidative stress can protect cardiac development under the exposure to isoniazid.

## Data Availability

All data generated and analyzed during this study are included in this article. The datasets used and/or analyzed during the current study are available from the corresponding author on reasonable request.

## Abbreviations

amhc: Atrial myosin heavy-chain
AO: Acridine orange
CAT: Catalase
cmlc2;: Cardiac myosin light chain 2
cTNT: Cardiac troponin T
hpf: Hours post-fertilization
MDA: Malondialdehyde
NAC: N-acetyl-L-cysteine
PBS: Phosphate-buffered saline
ROS: Reactive oxygen species
SOD: Superoxide dismutase
SSC: Saline sodium citrate
vmhc: Ventricular myosin heavy-chain
